# The Newcastle 85+ study: biological, clinical and psychosocial factors associated with healthy ageing: study protocol

**DOI:** 10.1186/1471-2318-7-14

**Published:** 2007-06-26

**Authors:** Joanna Collerton, Karen Barrass, John Bond, Martin Eccles, Carol Jagger, Oliver James, Carmen Martin-Ruiz, Louise Robinson, Thomas von Zglinicki, Tom Kirkwood

**Affiliations:** 1 Institute for Ageing and Health, Newcastle University, Newcastle upon Tyne, UK; 2 Department of Health Sciences, University of Leicester, Leicester, UK

## Abstract

**Background:**

The UK, like other developed countries, is experiencing a marked change in the age structure of its population characterised by increasing life expectancy and continuing growth in the older fraction of the population. There is remarkably little up-to-date information about the health of the *oldest old *(over 85 years), demographically the fastest growing section of the population. There is a need, from both a policy and scientific perspective, to describe in detail the health status of this population and the factors that influence individual health trajectories. For a very large proportion of medical conditions, age is the single largest risk factor. Gaining new knowledge about why aged cells and tissues are more vulnerable to pathology is likely to catalyse radical new insights and opportunities to intervene. The aims of the Newcastle 85+ Study are to expose the spectrum of health within an inception cohort of 800 85 year-olds; to examine health trajectories and outcomes as the cohort ages and their associations with underlying biological, medical and social factors; and to advance understanding of the biological nature of ageing.

**Methods:**

A cohort of 800 85 year olds from Newcastle and North Tyneside will be recruited at baseline and followed until the last participant has died. Eligible individuals will be *all *those who turn 85 during the year 2006 (i.e. born in 1921) and who are registered with a Newcastle or North Tyneside general practice. Participants will be visited in their current residence (own home or institution) by a research nurse at baseline, 18 months and 36 months. The assessment protocol entails a detailed multi-dimensional health assessment together with review of general practice medical records. Participants will be flagged with the NHS Central Register to provide details of the date and cause of death.

**Discussion:**

The Newcastle 85+ Study will address key questions about health and health-maintenance in the 85+ population, with a particular focus on quantitative assessment of factors underlying variability in health, and on the relationships between health, nutrition and biological markers of the fundamental processes of ageing.

## Background

The UK, like other developed countries, is experiencing a marked change in the age structure of its population, characterised by increasing life expectancy and continuing growth in the older fraction of the population [1-4]. Contrary to all major forecasts by national and international agencies over the last few decades, life expectancy has not reached a plateau but has continued to increase by about two years per decade [5-7]. In addition to the obvious reasons for the priority assigned to improving the health and care of older people within the UK through activities such as the NHS Strategic Review of Ageing and Age-Associated Disease and Disability [8] and the National Service Framework for Older People [9], there is great scientific interest in studying in detail what is happening to the health of the *oldest old *(over 85 years) and the factors that influence individual health trajectories. These insights are important, not only to inform us about the health status of what is demographically the fastest growing section of the population (about whom there is remarkably little up-to-date information), but also for what they will tell us about the ageing process itself and the factors that affect it. For a very large proportion of medical conditions, age is the single largest risk factor. Gaining new knowledge about why aged cells and tissues are more vulnerable to pathology is likely to catalyse radical new insights and opportunities to intervene. The rapidly declining mortality rates of the very old point to much greater intrinsic malleability in the mechanisms of ageing than has hitherto been appreciated. This fits well with new biological understanding of ageing, which has been led to a significant extent by our own previous research [10-15]; ageing is not programmed but results from a gradual, lifelong accumulation of subtle damage in the cells and tissues of the body over time. This is modulated by genetic factors, chiefly those involved in maintenance and repair (e.g. DNA repair, antioxidant defences). However, the process appears to be susceptible to a wide range of non-genetic factors, particularly nutrition and lifestyle, which in turn may be affected by socioeconomic status. Genes account for about 25% of the variability of human longevity [16], and the remaining 75% is believed to be due to factors such as nutrition, lifestyle, and socioeconomic variables, but as yet these have not been determined with any precision. This biological understanding is important for the emphasis it places on the life-course nature of the ageing process and its amenability to positive interventions. A striking feature of biological ageing is its marked variability between individuals [17-22]. This is particularly evident among the oldest old where some individuals preserve strikingly high levels of health and functional ability. Understanding the sources of variability in the oldest-old phenotype will be essential to identify factors contributing to malleability of the ageing process.

Very few measures of health status have been applied systematically within the 85+ age group in the UK [23-25] and no study has yet attempted a comprehensive assessment of biological, medical and social characteristics. The MRC Trial of Assessment and Management of Older People in the Community compared different methods of screening and management of older people, excluding those in long stay hospitals and care homes [26-30]. This mainly cross-sectional trial provided valuable baseline health information on 33,000 75+ year olds, including 5000 in the 85+ age group, with some follow-up for mortality and hospital/institutional admissions. Tinker et al [31], in an analysis of national survey data from the 1990s, compared the housing, general health and care use of people in the 85+ age group to those aged 65–84. The importance of socioeconomic factors for health of those still in employment was shown by the Whitehall Study [32]. The English Longitudinal Study on Ageing [33] is focusing on interactions between health and socioeconomic status in 11,000 individuals across a broader age range from 50 to 80+; however the information on the oldest old is relatively scarce with only 250 participants aged 85 and over at baseline and potential bias due to the sampling method used. Other previous studies relevant to understanding health among the very old are the MRC Cognitive Function and Ageing Study (CFAS) [34,35] and the Leiden 85+ Study [36,37]. CFAS is a population-based study, in six centres in England and Wales (including Newcastle), of people aged 65 or over living in the community, including long-term care institutions. It is a longitudinal study of 13,004 participants sampled in 1990, with the most recent follow up completed in 2003. At baseline, 1508 participants were aged 85 and over. The core aim of CFAS has been to estimate the prevalence and incidence of cognitive impairment and dementia. The most direct investigation of the overall health status of the very old is the Leiden 85+ Study which recruited 599 individuals aged 85 in 1997 (response rate 87%) and followed them, with annual visits, for five years. The Leiden study included measures on a range of health variables, with a particular focus on inflammation and vascular factors.

The Newcastle 85+ Study design has been developed and fully tested during a cross-sectional study of 85 year olds from four Newcastle general practices which was conducted in 2003–2004. Eighty-nine individuals were recruited to the full study (66% of those eligible) with an additional 27 (20% of those eligible) agreeing to review of general practice medical records only. The results and experience of this study helped shape the detailed plans for the main project. Extensive consultation has taken place with local consumer groups together with other stakeholders such as the local primary care trusts, general practitioners, community nursing staff, social services and care home managers. Close links have also been established with local media which will be used to publicise the study.

The Newcastle 85+ Study is designed using experience from the pilot study, from the Leiden group, with whom we have close collaboration, and with expertise gained within MRC CFAS. The Newcastle study will address key questions about health and health-maintenance in the 85+ population, with a particular focus on quantitative assessment of factors underlying variability in health and on the relationships between health, nutrition and biological markers of fundamental processes of ageing. It will include a range of important questions not included in the Leiden study, particularly with respect to detailed analyses of nutrition and biological markers of ageing.

### Aims

The aims of the Newcastle 85+ Study are to:

i). Expose the spectrum of health within an inception cohort of 800 85 year-olds, selected without regard to health status as far as is possible, establishing the distributions (especially the variability) of a broad range of health measures within the group.

ii). Examine, in unprecedented detail, health trajectories and outcomes as the cohort ages and their associations with underlying biological, medical and social factors. This longitudinal element will focus on factors contributing to maintenance of health in this age group and assess the predictive value of biological and other markers of health as measured at baseline.

iii). Advance understanding of the biological nature of ageing.

## Methods

**Figure 1:**
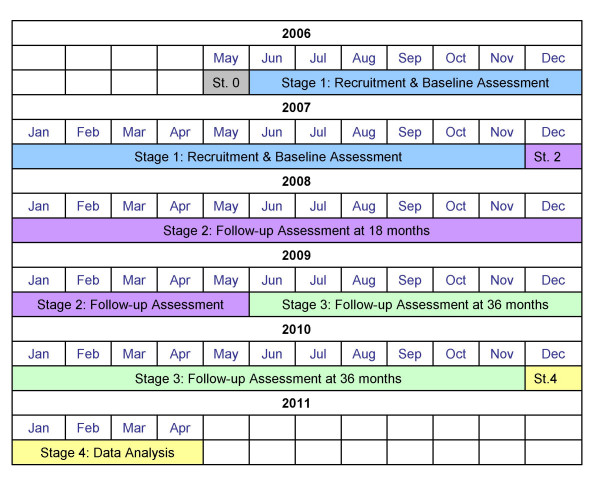
Gant chart of the Newcastle 85+ Study timelines.

A cohort of 800 85 year olds from Newcastle and North Tyneside will be recruited at baseline and followed until the last participant has died. The UK Medical Research Council, together with the Biotechnology and Biological Sciences Research Council, has provided funding for the first 5 years of the project. Ethical approval has been obtained from Newcastle & North Tyneside Local Research Ethics Committee. The first 5 years are divided into 4 stages as illustrated in Figure 1. The stages have been designed to maximise the length of follow-up whilst allowing key results to be obtained during the funded period.

### Stage 0: Staff training and piloting of assessment instruments (1 month)

### Stage 1: Baseline recruitment and assessment (18 months duration)

#### Recruitment

The cohort will be recruited from general practices in Newcastle and North Tyneside Primary Care Trusts. Eligible individuals will be *all *those who turn 85 during the year 2006 (i.e. born in 1921) and who are registered with any Newcastle or North Tyneside Primary Care Trust general practice. All individuals who meet these inclusion criteria will be invited to participate, whether living at home or in an institution, and regardless of their state of health (except for exclusion by their general practitioner (GP) due to end stage terminal illness). Potential participants will be identified through the primary care trusts' GP patient lists and we will liaise with GPs to cross-check these lists (to exclude recent deaths and the terminally ill) and to request a 'letter of support'.

The study team will send each potential participant a letter of invitation together with an information pack, photographs of those research staff involved in home visits and a 'letter of support' from their GP. The letter of invitation will be followed after one week by a telephone call or home visit from a research nurse to discuss the study in more detail. Previous experience in similar studies involving very old people has shown that direct telephone contact or visiting are the only reliable ways of finding out whether individuals are interested in participating in research. As many very old people are frail, they may find posting reply slips or making the first telephone call themselves too much of a burden. No pressure is put on the person to participate; this procedure has been adopted purely as a way of ensuring the older person receives all the relevant information to enable them to make an informed decision about participation. Once an individual has shown an interest in participating, an appointment is made to visit them in their own home. The purpose of this visit is to ensure that they have received and understand the study information and the implications of their involvement. If they wish to participate, written informed consent is then obtained.

As around 15% [34] of the proposed sample may have significant cognitive impairment, due consideration has been given to the ethical issues this raises. It is important not to exclude such people from scientific research as, so doing, could prevent this group from benefiting from advances in medical understanding. However there is a need to carefully consider the consent process. The Mental Capacity Act 2005 provides a statutory framework 'to empower and protect vulnerable people who may not be able to make their own decisions' [38]. At the forefront of this framework is the principle that capacity of an individual to consent must be assumed unless it is proved otherwise. During the first visit to a potential participant, the research nurse will establish their level of capacity utilising a consent checklist and consent pathway, based upon information from the individual, family and significant others. Each participant will be asked to give consent appropriate to their level of understanding ranging from written full informed consent to verbal or non-verbal communication, of which account will be taken in determining willingness to participate. In those individuals found to be without capacity to give full informed consent, proxy assent/consultee approval will also be sought from a close relative or, in those potential participants without relatives, from an immediate carer. They will be asked to use their knowledge of the participant, in the past and the present, and will be required to state that, in their opinion, the participant would have had no objection to entering the study when cognitively intact, and would not be caused undue distress by participation. The consent protocol incorporates the notion that consent is a process and as such requires ongoing confirmation prior to and within each contact. If a participant is found to have lost capacity during the course of the study, consent is not deemed as being enduring and a process of gaining proxy assent/consultee approval is initiated, whilst suspending all contacts.

#### Assessment

Participants will be visited in their current residence (own home or institution) by a research nurse. If a potential participant is temporarily in hospital at the time of recruitment (1% of those eligible to participate in the pilot study), assessment will be deferred until discharge. Home based assessment is vital for high recruitment levels in this age group; in our pilot study over half of the sample would have been unwilling to attend hospital for assessment.

The baseline assessment protocol entails a detailed multi-dimensional health assessment by means of:

a). Questionnaires: socio-economic status; family data; physical health (global health status, longstanding illness, angina, shortness of breath, falls, generalised pain, joint pain, fractures, incontinence, vision and hearing); psychological health (depression); disability; diet; oral health; lifestyle (smoking, alcohol and exercise); social support and participation; use of health and social care.

b). Measurements and function tests: weight, bio-impedance (body composition-fat and water), demi-span, waist and hip circumference, tooth count, blood pressure, hand-grip strength, walking test (timed 'up and go' test), cognitive function (mini-mental state examination and computerised assessment of memory and attention (CDR battery)), electrocardiogram, spirometry and oximetry.

c). Blood tests: blood samples, taken after an overnight fast where possible, will be analysed for:

1. *Routine haematology and biochemistry*: full blood count; creatinine and electrolytes; liver panel; bone panel; glucose; glycosylated haemoglobin.

2. *Lipid profile*: cholesterol, triglycerides, high and low density lipoproteins, apolipoproteins (A1 and B).

3. *Thyroid function*: free T4, free T3, reverse T3, TSH and TPO antibodies.

4. *Inflammatory markers*: High sensitivity CRP, rheumatoid factor, cytokines (TNF-alpha and Interleukin 6).

5. *Cortisol*

6. *Nutritional markers*: Vitamins B2, B6, B12, C and D, ferritin, red cell folate and homocysteine.

7. *Biomarkers*: DNA repair capacity, telomere length, F2-isoprostane (marker of oxidative stress).

8. *Markers of immunosenescence*: T cell oligoclonality and lymphocyte subpopulation distributions (senescent T-cells, memory T-cells and NKcells).

In addition, DNA, RNA and plasma will be stored for future analyses (including genetic, proteomic, metabolomic and allostatic load markers concerned with ageing [39]).

Information will be gathered during three nurse visits each lasting about one hour and 30 minutes and one additional nurse visit to collect the fasting blood samples; visits to individual participants will take place over one month. Data will be entered directly onto a tablet style laptop computer. For those participants who are particularly frail, there will be the option to spread the assessments over more visits, to have a shorter assessment in total and for much of the interview to be conducted with an 'informant' i.e. a relative/carer who knows the participant well. With participants' consent, their GP will be informed about their participation and of any abnormal assessment findings of immediate clinical relevance. In addition participants' GP records will be reviewed for information on medication, key diagnoses and use of GP services. For those individuals who decline to participate in the full assessment, there will be the option to consent to review of records only.

The full protocol was tested during the pilot study and shown to be fully practical and practicable. The schedule was shown to be acceptable to participants; 96% rated their involvement in the study as positive or very positive and 93% of those recruited completed all the nurse visits. Safety issues have been addressed; research nurses will have enhanced Criminal Records Bureau clearance and carry photo-identification on visits, participants will be sent photographs of the research team with their invitation letter and local police will be informed about the study. For staff safety, the location of staff involved in community visits will be monitored using an automated telephone call-back system.

It will not be possible to check inter-rater reliability for home assessment data collection due to the participant interview burden this would entail. However reliability of the data extraction from GP records will be examined in a core set of variables which cover the range of difficulty and modes of extraction from GP records. From the pilot data, where 3 individuals extracted data independently from the same GP records, we found the intraclass correlation coefficient (ICC) to vary from 0.55 for number of heart attacks to 0.99 for number of consultations. We estimate therefore that 9 nurses extracting information from the same set of 32 notes will have 80% power with a type I error rate of 5% to show at least moderate agreement (ICC>0.4) for the majority of variables extracted.

### Stages 2 and 3: Follow-up assessments at 18 months and 36 months (each of 18 months duration)

It is anticipated that 9–13% of the cohort will die per year with additional cohort loss due to drop out of 5–10% per year (Gussekloo, J. 2004, personal communication); increasing age is a key predictor of attrition (and non-response) in population studies of older people [40]. Participants will be flagged with the NHS Central Register to provide details of the date and cause of death. Survivors will be revisited in Stage 2 (18 months from baseline) and Stage 3 (36 months from baseline). The exact detail of the assessments to be conducted at follow-up has yet to be decided. It is envisaged that there will be a core assessment comprising:

a). Questionnaires: changes in living arrangements, physical health, psychological health, disability, lifestyle, social support and participation and use of health and social care.

b). Measurements and function tests: weight, blood pressure, hand-grip strength, walking test and cognitive function.

c). Blood samples: routine haematology and biochemistry; lipid profile; inflammatory markers; biomarkers of ageing (telomere length, oxidative stress, immunosensecence) and storage of samples for future analyses (RNA, proteomics, metabolomics and allostatic load markers).

A repeat review of GP records will be conducted in Stage 3 to obtain information on key medical events occurring during the follow-up period. In addition, additional funding will be sought to allow further key domains of health to be addressed in more detail including nutrition, oral health, cardiac disease and musculoskeletal disease.

### Sample size considerations

Since there are a large number of specific factors to be analysed, formal sample size calculations for the study as a whole were not feasible.

The number was decided upon from the following considerations:

(i) A previous study of similar design (Leiden 85+ Study) had 599 participants, which proved sufficient for a range of important, statistically significant conclusions to be drawn. We have increased this by one third.

(ii) Based on the pilot study, this is the number we can expect to recruit from those turning 85 within a single year inside a study area of manageable extent, taking account of the need to return blood samples within a defined short window of time for some of the laboratory analyses.

(iii) Statistical calculations indicated that, based on data from the pilot study, this sample size will deliver appropriately precise estimates of the main effects within major sub-groups.

### Stage 4: Data analysis

Interim analyses will take place after the completion of each phase. In Stage 4, full analysis of the data will be conducted using multi-variate statistical methods and incorporating systems biology modelling approaches which make use of advanced mathematical and computational techniques.

## Discussion

The Newcastle 85+ Study will address key questions about health and health-maintenance in the 85+ population, with a particular focus on quantitative assessment of factors underlying variability in health, and on the relationships between health, nutrition and biological markers of the fundamental processes of ageing. Given the age of the participants, we expect significant morbidity and mortality among the cohort (life expectancy at age 85 is 5 years for men and 6 years for women [41]) and we will be able to analyse how these various outcomes can be related to measures recorded at baseline and during follow-up. One of the main objectives of the study is to identify factors associated with the different health trajectories followed by individuals after age 85. There is pronounced variability in individual ageing trajectories for reasons which are at present poorly understood. By conducting regular follow-ups we will be able to identify the variability in individual health trajectories within the cohort and to examine their associations with biological and other factors. The focus will be on factors involved in the *maintenance *of health among those who have already attained age 85, not on those affecting survival to 85. Although the latter is of great interest, extensive data already exist on factors influencing morbidity and mortality in middle and young-old age groups, whereas there are hardly any data on health maintenance among the oldest old. Given the rapid increase in this age group and their risk of high dependency, identification of factors that can maintain healthy, independent living are of primary interest in their own right, quite apart from what they can tell us about the ageing process.

To address the full complexity of genetic and non-genetic factors affecting healthy ageing across the full range of geographical and ethnic diversity within the UK would be prohibitively extensive and expensive. We have chosen a single age at recruitment and a single geographical region in order to capture as clear a picture as possible of the factors explaining variability *within *this cohort. If we can clearly identify factors associated with maintenance of health in 85-year old Newcastle/North Tyneside residents, not only will this help us to understand the interplay of such factors with underlying processes of ageing themselves, but it is highly likely that the actions of such factors will play out in similar ways in other populations. This can be verified and future studies streamlined using the Newcastle 85+ Study as a reference point. A particular benefit of the population in North East England is that it shows exceptional genetic and social stability, while also covering all socioeconomic groups.

We recognise that what we are proposing is different from the hypothesis-driven approach that has characterised most epidemiological and clinical trial work to date, yet we are firmly convinced that such an approach is not only timely but necessary to begin to answer some fundamentally important questions about the ageing process, which will have major long-term relevance for public health not only of the oldest old. In effect, what we propose is a systems-biology approach to human ageing. Many areas of the life sciences, including biomedicine, are moving towards recognition that such approaches, which are characterised by using data to develop predictive models that contribute new information and suggest hypotheses for further tests, are essential to understand complex phenomena.

## Competing interests

As noted below, the pilot study was partly supported by a gift from Unilever Corporate Research.

## Authors' contributions

All authors were substantially involved in the conception and design of this study. JC and TK drafted the manuscript with all other authors contributing to its critical review and approving the final draft.

## Pre-publication history

The pre-publication history for this paper can be accessed here:


